# Potential Metabolic Biomarkers for Early Detection of Oral Lichen Planus, a Precancerous Lesion

**DOI:** 10.3389/fphar.2020.603899

**Published:** 2020-11-06

**Authors:** Xiao-Shuang Wang, Zhi Sun, Li-Wei Liu, Qiu-Zheng Du, Zhang-Suo Liu, Yan-Jie Yang, Peng Xue, Hong-Yu Zhao

**Affiliations:** ^1^ Stomatological Hospital of Henan Province, The First Afﬁliated Hospital of Zhengzhou University, Zhengzhou, China; ^2^ School and Hospital of Stomatology of Zhengzhou University, Zhengzhou, China; ^3^ Department of Pharmacy, The First Afﬁliated Hospital of Zhengzhou University, Zhengzhou, China; ^4^ Henan Key Laboratory of Precision Clinical Pharmacy, Zhengzhou, China; ^5^ Department of Nephrology, The First Affiliated Hospital of Zhengzhou University, Zhengzhou, China; ^6^ Health Management Centre, The First Afﬁliated Hospital of Zhengzhou University, Zhengzhou, China

**Keywords:** oral lichen planus, UHPLC-Q-orbitrap HRMS, metabolomics, precancerous lesion, biomarkers

## Abstract

**Background:** Oral lichen planus (OLP) is a T-cell-mediated chronic inflammatory disorder and precancerous oral lesion with high incidence. The current diagnostic method of OLP is very limited and metabolomics may provide a new approach for quantitative evaluation.

**Methods:** The Ultra-Performance Liquid Chromatography-Quadrupole/Orbitrap High Resolution Mass Spectrometry (UHPLC-Q-Orbitrap HRMS) was applied to analyze the change of metabolites in serum of patients with OLP. A total of 115 OLP patients and 124 healthy controls were assigned to either a training set (*n* = 160) or a test set (*n* = 79). The potential biomarkers and the change of serum metabolites were profiled and evaluated by multivariate analysis.

**Results:** Totally, 23 differential metabolites were identified in the training set between OLP group and healthy group. Three prominent metabolites in receiver operating characteristic (ROC) were selected as a panel to distinguish OLP or healthy individuals in the test set, and the diagnostic accuracy was 86.1%.

**Conclusions:** This study established a new method for the early detection of OLP by analyzing serum metabolomics using UHPLC-Q-Orbitrap HRMS, which will help in understanding the pathological processes of OLP and identifying precancerous lesions in oral cavity.

## Introduction

Oral lichen planus (OLP) is a T-cell-mediated chronic inflammatory disorder and potentially precancerous oral lesion, with a prevalence of 0.5–3% (Warnakulasuriya et al., 2007; Carbone et al., 2009; Kountakis, 2013). About 0.5–12.5% of patients with OLP will progress to malignant transformation (Kountakis, 2013). The diagnosis of OLP is usually based on dental exam, histological examinations and patient history (Alrashdan et al., 2016; Raj and Patil, 2017). Due to the lack of universal diagnostic criteria for OLP and related lesions such as oral epithelial dysplasia (OED), OLP with dysplasia and oral lichenoid lesions (OLL), misdiagnosis often occurs, leading to the incorrect treatment strategy (van der Meij et al., 1999; Au et al., 2013; Raj and Patil, 2017). Therefore, a more efficient, non-invasive and accurate diagnostic method for OLP is urgently needed.

As one of the major components of system biology, metabolomics is a well-established method to assess global metabolic profiles through biomarker discovery in accessible biofluids (Wang et al., 2012; Zhang et al., 2014; Yang et al., 2018). It shows great potential as a way of identifying biomarkers for various diseases (Tohge and Fernie, 2010). In recent years, the application of metabolomics in the diagnosis, etiology, prevention and treatment of oral diseases has attracted attentions in the field of stomatology (Rai et al., 2018). In 2017, Yang *et al.*(Yang et al., 2017)used metabolomics to detect plasma metabolites in 20 erosive OLP patients and successfully found ten differential biomarkers. Subsequently, the urinary metabolome was analyzed and 12 kinds of metabolites were found to have changed (Li et al., 2017). Recently, 16 oral epithelial tissue samples from reticular OLP patients were detected by metabolomics. As a result, 21 metabolites and eight signaling pathways were identified (Yang et al., 2018). These studies provided important references for further understanding the pathogenesis of OLP. However, due to the small sample size and other limitations in studies, further experimental and clinical researches are still needed.

In this study, ultra-performance liquid chromatography-quadrupole/orbitrap high resolution mass spectrometry (UHPLC-Q-Orbitrap HRMS) was used for metabolomic analysis. Serum samples from 160 healthy subjects and OLP patients were assigned as a training set to identify potential biomarkers for the early detection of OLP. To confirm the reliability and accuracy, an independent test sample set (*n* = 79) was used to evaluate the biomarkers panel we identified.

## Materials and Methods

### Instruments and Reagents

UHPLC-Q-Orbitrap System: Ultimate 3000 UHPLC (Dionex, United States), Q Exactive high resolution mass spectrometry (Thermo Fisher Scientific, United States); ACQUITY UHPLC^®^ BEH C_18_ (100 × 2.1 mm, 1.7 µm) chromatographic column (Waters, United States); AL104 balance with 0.0001 accuracy (Mettler Toledo, Switzerland); Centrifuge CF16RN (HITACHI, Japan); HeraeusFresco17 (Thermo, United States); Acetonitrile and methanol (UPLC-grade, Fisher Scientific, United States); formic acid (UPLC-grade, Aladdin Industrial Co., Ltd., China); Internal standard (L-2-chlorophenylalanine and ketoprofen) (Sigma, United States; J&K Chemical, China); Milli-Q water purification system (Millipore, Shanghai, China); all solutions were filtrated by 0.22 µm pore size filters.

### Participants

A total of 239 participants were recruited including 115 OLP patients (100 patients with reticular OLP and 15 patients with erosive OLP) and 124 healthy individuals from March 1, 2019 to August 30, 2019 at the First Affiliated Hospital of Zhengzhou University. Patients were randomly assigned (by a random-number Generator in Excel) to either a training set (*n* = 160) or a test set (*n* = 79). The distributions of gender, age and other information were listed in Table 1. The written informed consent was obtained from all participants. The approval of the Ethical Committees of the First Affiliated Hospital of Zhengzhou University (Name of IRB: Ethics Committee of Scientific Research Project of the First Affiliated Hospital of Zhengzhou University; Date of approval: February 26, 2019; Ethical number: 2019-KY-26) were obtained. This research was conducted in accordance to the ethical guidelines of the 1975 Declaration of Helsinki.

**TABLE 1:** The baseline characteristics of 239 participants.

	OLP (*n* = 115)	Control (*n* = 124)	*p*
Age (years)	50.66 ± 13.73	47.66 ± 13.23	0.870
Gender (male/female)	48/67	56/68	0.596
BMI (kg/m^2^)	24.32 ± 3.79	23.35 ± 3.46	0.059
Smoking history**	50/115	9/124	6.90E−11
Drinking history	25/115	18/124	0.150
Prefer spicy food	45/115	41/124	0.330
Prefer very hot food**	51/115	21/124	3.50E−06
Lack of exercise**	85/115	32/124	3.13E−15
Poor sleep*	19/115	9/124	0.028

Data are presented as the mean ± SD. BMI, body mass index; Smoking history: ≥1 cigarette per day for more than half a year; Drinking history: drinking alcohol more than once a week (alcohol content ≥50 ml) for more than half a year; Prefer very hot food: often intake high-temperature-treated meals within 1 min; Lack of exercise: exercise less than once a week; Poor sleep: sleep less than 6 h per night. *p < 0.05, **p < 0.001, ***p < 0.001, student’s *t*-test.

The diagnosis of OLP was made by one oral clinician based on the clinical and pathological criteria and independently confirmed by two pathologists. Excluding criteria: 1) Received any treatment prior to admission; 2) Had other systemic diseases such as cardiovascular diseases, hypertension, kidney disease, diabetes, and other intraoral inflammation, etc.; and 3) Used antibiotics, hormones, or immunomodulator for at least 3 months.

### Sample Collection and Preparation

Blood samples were collected in the morning after fasting at patients’ initial visit (Walsh et al., 2006). Samples were put in vacutainer tubes containing coagulant, which is a silica gel blood coagulation activator to promote the coagulation of blood samples, and cooled down in the insulated ice packs, then transferred to the laboratory immediately within half an hour. They were centrifuged at 3,000 × *g* for 10 min at 4°C. Supernatants (serum) were separated and transferred into new vials, and immediately cryopreserved at −80°C until used.

After thawing on ice, the serum (100 µl) sample was added into 300 µl methanol solution (containing 0.05 μg/ml L-2-chlorophenylalanine and 0.5 μg/ml ketoprofen as internal standard). After vortexing for 1 min, the mixture was centrifuged at 16,200 × *g* at 4°C for 10 min 200 μl pipette gun was used to suck out the supernatant and transferred to an auto-sampler vial for analysis.

In order to ensure the reliability, quality control (QC) sample analysis was carried out in the process of metabolomics data collection. Six QC samples were first analyzed, and sample analysis was started after the instrument was stabilized. After that, QC samples were evenly inserted every ten samples in the sequence of sample analysis to monitor the stability of analysis. Blank samples containing only solvent are inserted after each QC sample to avoid contamination.

### UHPLC-MS/MS System Conditions

An ultra high performance liquid chromatography (UHPLC) system was used to separate the metabolites in serum. Five microliters aliquot from each sample was injected into a ACQUITY UHPLC^®^ BEH C_18_ column maintained at 40°C. The mobile phase was the acetonitrile (A) with 0.1% formic acid aqueous solution (B). The gradient elution was as follows: at a flow rate of 0.2 ml/min: 0–0.5 min, 5% A; 0.5–1.0 min, 5–60% A; 1.0–7.0 min, 60–80% A; 7.0–9.0 min, 80–100% A; 9.0–11.0 min, 100% A; 11.0–11.2 min, 100–5% A; 11.2–13.0 min, 5% A.

A Q Exactive high resolution mass spectrometry was tandem to the UHPLC system using a heated electrospray ionization (HESI) source. The temperature of the auxiliary gas, ion source, and capillary were 300°C, 350°C, and 320°C, respectively, the flow rate of the auxiliary gas was 10 arb. Samples were respectively tested in the positive and negative modes by full scan/ddms two scan patterns from 80 to 1,200 *m/z* at the mass resolving power of 17,500 in MS/MS. The gradient collision energy was at 20, 40, and 60 eV. The spray voltage and the sheath gas flow rate were set to 3.50 kV and 40 arb for the positive mode and 2.80 kV and 38 arb for the negative mode. The order of sample analysis was randomized.

### Data Processing and Statistical Analysis

All data were acquired and processed by Thermo Xcalibur^TM^ software (Version 3.0, Thermo Scientific, United States). Then the peak calibration, peak matching and peak alignment were performed by the Compound Discovery software (Version 3.0, Thermo Scientific) to extract the information. Specific parameters were as follows: the width of Retention Time (RT) is set to 0.1 min and the mass width to 5 ppm. The intensity threshold for filtering ion peak is 1,000,000 in both the positive ion mode and negative ion mode. The data result set which corresponds to the concentration of certain metabolite, contained all the *m/z* value, RT and ion peak area of each sample. And they were exported to the multivariate statistical software SIMCA (version 14.0, Umetrics, Umea, Sweden) for the subsequent principal component analysis (PCA) and orthogonal partial least square discrimination analysis (OPLS-DA), and Variable importance in projection (VIP) was obtained from the OPLS-DA model. A 200 times permutation test was performed to assess the risk of overfitting for the model. The preliminary discriminating metabolites were selected by plotting the data set of all the discriminators in a volcano plot. To further screen the significant variables between the two groups, a student’s *t*-test and fold change of all the detected peaks were carried out by the SPSS 21.0 software (IBM, United States). Finally, the metabolites with statistical significance were selected and identified to distinguish the difference between the two groups. A Heatmap was generated with these screened metabolites by MetaboAnalyst (https://www.metaboanalyst.ca/) to show the trend of change. For each identified differential metabolite, receiver operating characteristic (ROC) curve was drawn and areas under the curve (AUC) were calculated using the SPSS 21.0 software (IBM, United States).

In order to adjust the influence of confounding factors (in Table 1) on each differential metabolite, SPSS 21.0 software (IBM, United States) was used to establish a binary logistic regression model by taking groups as dependent variables and each metabolite and each confounding factor as covariables.

### Identification of Differential Metabolites

Compound identification was achieved by comparing the precision *m/z*, MS^2^, RT and other information collected by mass spectrometry with authentic standards or searching the Human Metabolome database (http://hmdb.ca/), the PubChem compound database.

### Metabolic Pathway Analysis and Pathological Network Construction

Metabolic pathway analysis of differential metabolites was performed by MetaboAnalyst based on the database source including The Human Metabolome database (HMDB), PubChem and Kyoto Encyclopedia of Genes and Genomes (KEGG).

## Results

### Demographic Characteristics

The baseline characteristics of these 239 participants was showed in Table 1. There were more cases had the habit of smoking (*p* < 0.001) and a preference for very hot food in the OLP group compared with the healthy subjects (*p* < 0.001). There was no significant difference among OLP and healthy controls in terms of the habit of drinking (*p* = 0.150) and the preference for spicy food (*p* = 0.330). In addition, most of the subjects in the OLP group lacked exercises (*p* < 0.001), and most of them did not get enough time for sleeping (*p* = 0.028).

Supplementary Figure S1 showed the binary logistic regression model established by taking groups as dependent variables and each metabolite and each confounding factor as covariables, in order to adjust the influence of confounding factors on each differential metabolite. It showed that 23 differential metabolites have strong correlation with the occurrence of OLP after adjusting for various risk factors.

### Data Quality Evaluation in Metabolomic Analysis

**FIGURE 1:**
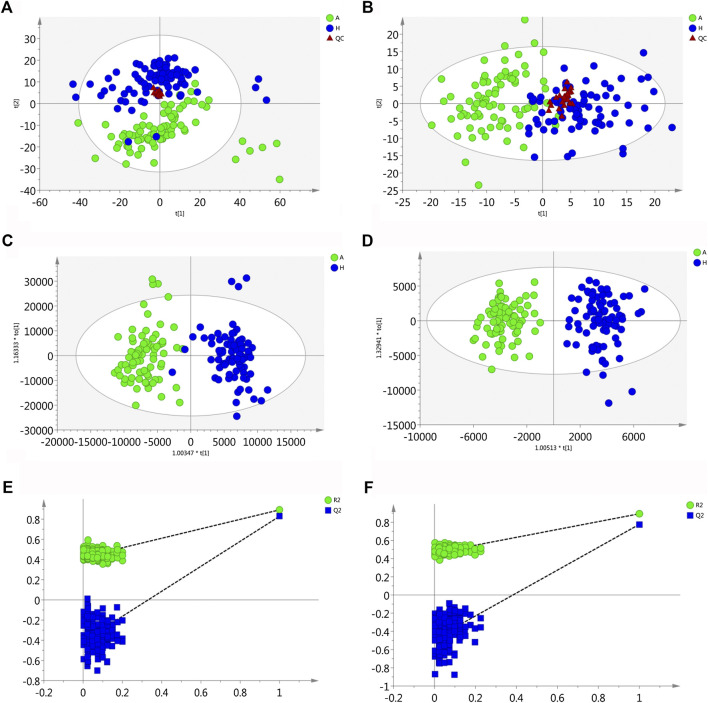
Multivariate statistical analysis of two groups. The principal component analysis (PCA) plot of QC and samples in **(A)** positive ion mode and **(B)** negative ion mode in the training set. The orthogonal partial least square discrimination analysis (OPLS-DA) score plots of OLP group vs. healthy control group in **(C)** positive ion mode and **(D)** negative ion mode in the training set. Cross-validation plot with a permutation test repeated 200 times of OLP group vs. healthy group in **(E)** positive ion mode and **(F)** negative ion mode in the training set. A, OLP group; H, healthy control; QC, quality control.

QC samples in both positive and negative mode were clustered closely in PCA score plots (Figures 1A,B), which indicated that the analytical process was stable and reliable.

### Metabolomics Analysis and Biomarkers Identification

**FIGURE 2:**
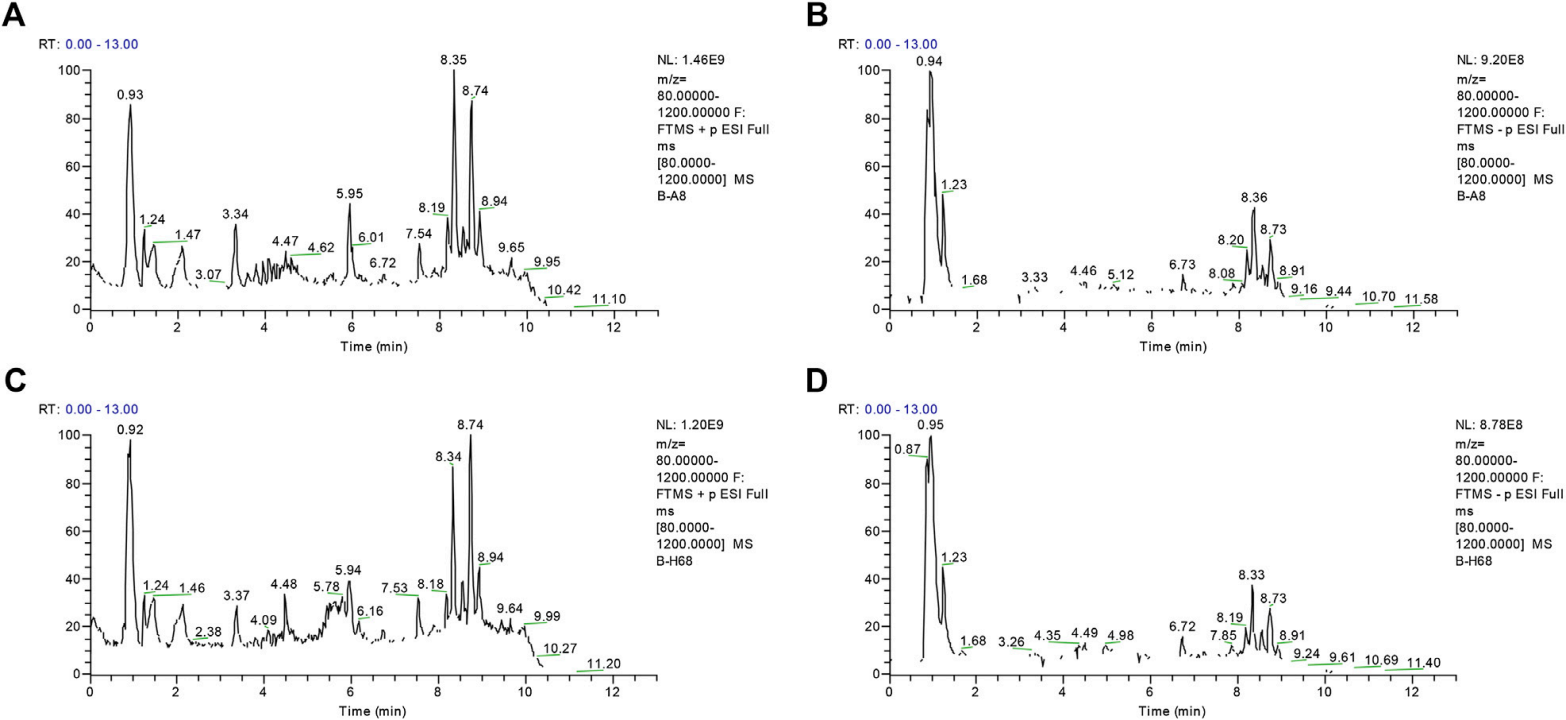
The representative total intensity chromatograms (TIC) of OLP and healthy group serum samples. TIC of OLP group obtained from the training set in **(A)** positive mode and **(B)** negative mode. TIC of healthy control obtained from the training set in **(C)** positive mode and **(D)** negative mode.

**FIGURE 3:**
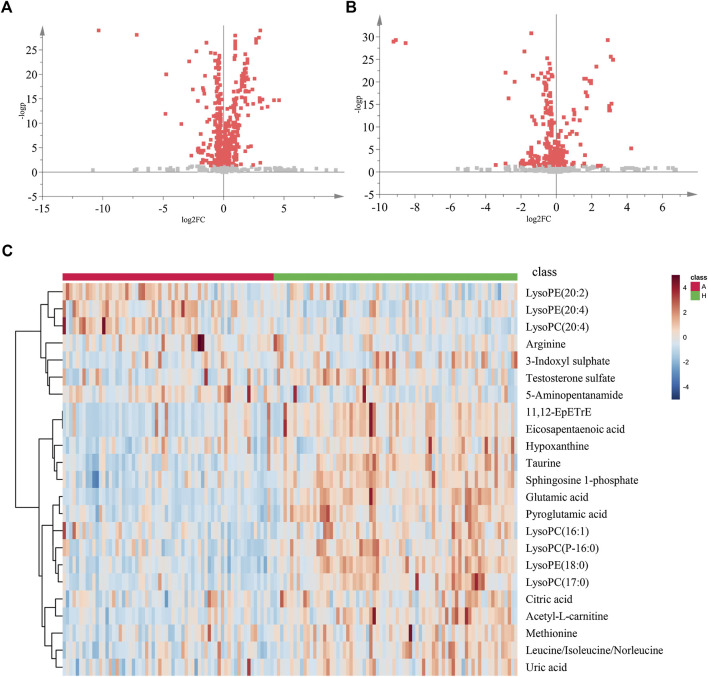
The volcano plot and heat map of two groups. The volcano plot of the OLP group vs. healthy group in **(A)** positive ion mode and in **(B)** negative ion mode. **(C)** The heat map of the changed metabolites in OLP group vs. healthy control. A, OLP group; H, health control.

Using the alignment software, 1,540 ion peaks in positive and 837 in negative mode and their areas were captured. The total intensity chromatograms (TIC) of serum samples obtained from the training set in both positive and negative ion modes are shown in Figure 2, which included the retention time, precise mass and ion intensity of the detected metabolites. Both PCA and OPLS-DA score plots in training set showed difference of metabolites between the OLP and control groups (Figures 1A–D). R^2^Y at 0.893 and Q^2^ at 0.777 in positive ion mode and R^2^Y at 0.892 and Q^2^ at 0.828 in the negative ion mode, suggesting that biochemical changes occurred in the blood of patients. The 200 times permutation test in both positive and negative ion modes (Figures 1E,F) demonstrated that the model was not overfitting (R^2^ at 0.423 and Q^2^ at −0.41 in positive in mode, R^2^ at 0.461 and Q^2^ at −0.482 in negative in mode). A volcano plot containing the *p* values of student’s t-test and fold changes was performed between the two groups to identify the differential metabolites (Figures 3A,B). In the volcano plot, the red dots represented the metabolites with the *p* values lower than 0.05 (–log_10_ *p* > 1.30). In this way, several potential biomarkers were distinguished which showed significant difference between the OLP and healthy group. Heat map of the Pearson correlation coefficients between differential metabolites and groups (Figure 3C) also revealed the distinct difference observed between the OLP and control groups. The metabolites with VIP values >1.0 and *p* values <0.05 for each comparison were shown in Table 2.

**TABLE 2:** Statistical analysis of potential metabolic biomarkers.

Id	Name	Formula	Retention time (min)	Molecular	VIP	Fold change	*p*	AUC
1	Glutamic acid	C_5_H_9_NO_4_	0.992	147.053	2.554	0.551	1.10E−22	0.916
2	LysoPE (18:0)	C_23_H_48_NO_7_P	8.749	481.317	8.845	0.826	3.47E−22	0.893
3	Taurine	C_2_H_7_NO_3_S	0.970	125.014	1.844	0.652	5.98E−15	0.850
4	LysoPC (17:0)	C_25_H_52_NO_7_P	9.199	509.347	1.424	0.676	1.22E−13	0.847
5	Sphingosine 1-phosphate	C_18_H_38_NO_5_P	7.786	379.248	1.090	0.788	1.21E−14	0.837
6	11,12-EpETrE	C_20_H_32_O_3_	8.729	320.235	2.269	0.442	2.96E−12	0.834
7	Eicosapentaenoic acid	C_20_H_30_O_2_	8.727	302.224	1.296	0.467	1.77E−11	0.819
8	Pyroglutamic acid	C_5_H_7_NO_3_	1.253	129.042	1.659	0.778	1.19E−11	0.815
9	LysoPC (P-16:0)	C_24_H_50_NO_6_P	8.973	479.336	1.150	0.757	1.05E−11	0.798
10	Acetyl-l-carnitine	C_9_H_17_NO_4_	1.247	203.115	1.604	0.666	4.06E−09	0.773
11	Leucine/Isoleucine/Norleucine	C_6_H_13_NO_2_	1.463	131.094	6.792	0.846	5.91E−07	0.730
12	5-Aminopentanamide	C_5_H_12_N_2_O	3.706	116.095	1.580	1.410	2.25E−04	0.719
13	LysoPC (20:4)	C_28_H_50_NO_7_P	8.336	543.331	4.907	1.278	7.46E−06	0.704
14	LysoPC (16:1)	C_24_H_48_NO_7_P	8.096	493.316	1.821	0.808	8.20E−06	0.698
15	LysoPE (20:2)	C_25_H_48_NO_7_P	8.360	505.316	4.235	1.125	9.93E−05	0.690
16	Methionine	C_5_H_11_NO_2_S	1.253	149.051	1.097	0.869	6.15E−04	0.683
17	Uric acid	C_5_H_4_N_4_O_3_	1.247	168.028	1.698	0.873	0.001	0.668
18	LysoPE (20:4)	C_25_H_44_NO_7_P	8.317	501.285	1.074	1.179	1.41E−04	0.668
19	Testosterone sulfate	C_19_H_28_O_5_S	8.194	368.165	2.104	0.724	0.016	0.660
20	Hypoxanthine	C_5_H_4_N_4_O	1.251	136.038	1.188	0.866	0.004	0.656
21	Citric acid	C_6_H_8_O_7_	1.256	192.027	1.868	0.883	0.002	0.619
22	3-Indoxyl sulfate	C_8_H_7_NO_4_S	4.818	213.009	1.934	0.710	0.014	0.604
23	Arginine	C_6_H_14_N_4_O_2_	0.884	174.111	1.238	1.123	0.031	0.567

### Establishment and Evaluation of the Metabolic Biomarkers Panel

**FIGURE 4:**
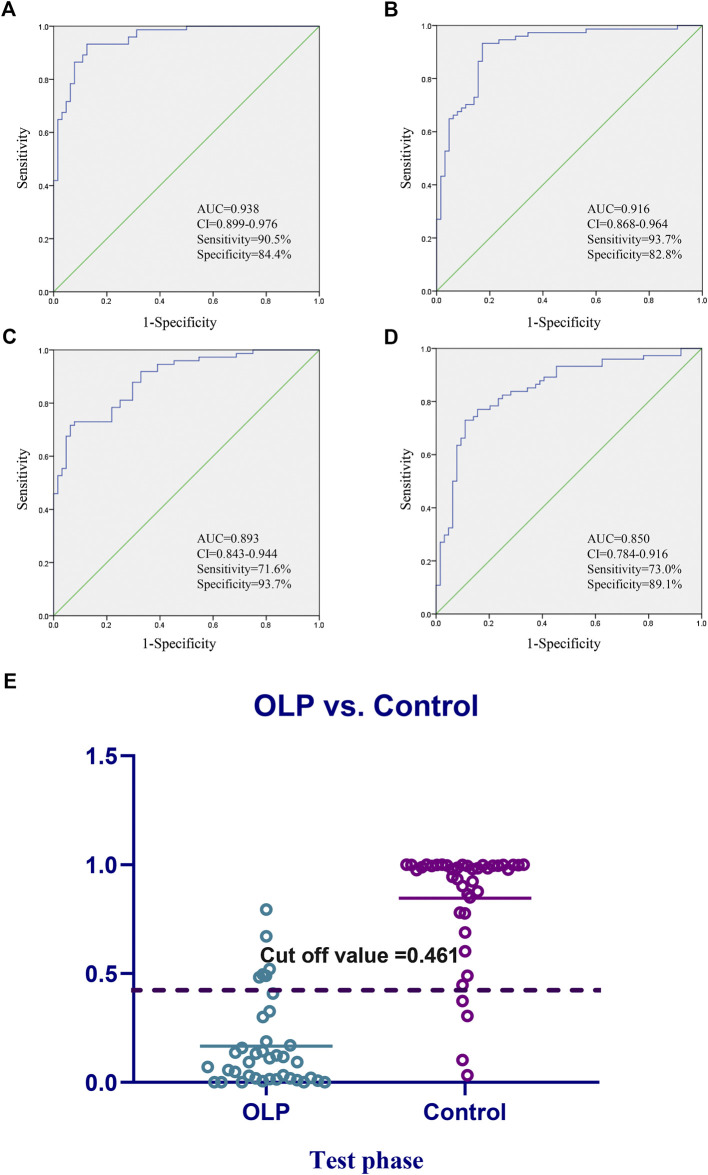
Diagnostic outcomes and prediction accuracies. Receiver operating curves (ROC) obtained by the **(A)** biomarkers panel, and **(B–D)** three highest metabolites in the comparisons between OLP group vs. healthy group. **(E)** The prediction accuracies by the biomarkers in test phase compared between OLP group vs. healthy group. AUC, area under curve; 95% CI, 95% confidence interval.

Considering the clinical significance of the identified differential metabolites, the combination of Glutamic acid, LysoPE (18:0), Taurine was proposed to be a panel of metabolic biomarkers for the early detection of OLP in patients. The ROC presentations appear in the logistic regression of the metabolic biomarkers panel from the training set (Figures 4A–D), and the test set (Supplementary Figure S2). The AUC, sensitivity, and specificity are 0.938, 90.5%, and 84.4%, respectively. Supplementary Figure S3 showed the structure and mass fragmentation pattern of these three metabolic biomarkers and mass fragmentation pattern in real samples. The mass fragmentation pattern from the peak in real sample was matched with the pattern of these three metabolic biomarkers.

On the basis of the highest prediction sensitivity and specificity of the ROC in the training set, the optimal cut-off value was 0.461 for OLP and healthy control groups. This cut-off value was then used to distinguish different individuals in the test set and the diagnostic accuracy was 86.1% (Figure 4E).

### Metabolic Pathway Analysis of Potential Biomarkers

**FIGURE 5:**
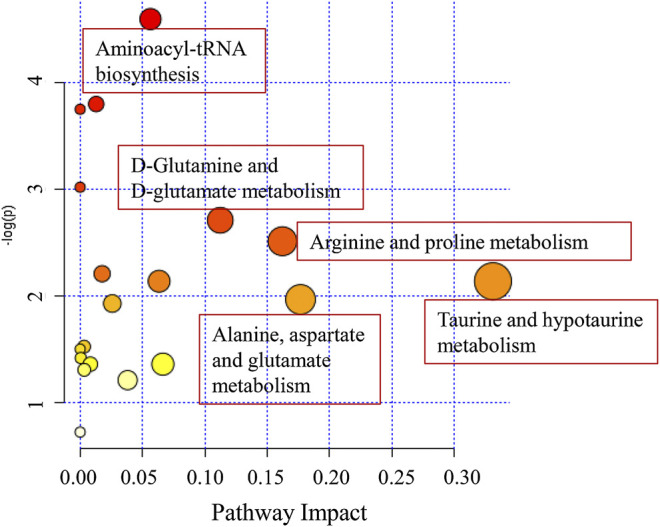
The disturbed metabolic pathways showed various metabolism changes between OLP group and healthy control.

**FIGURE 6:**
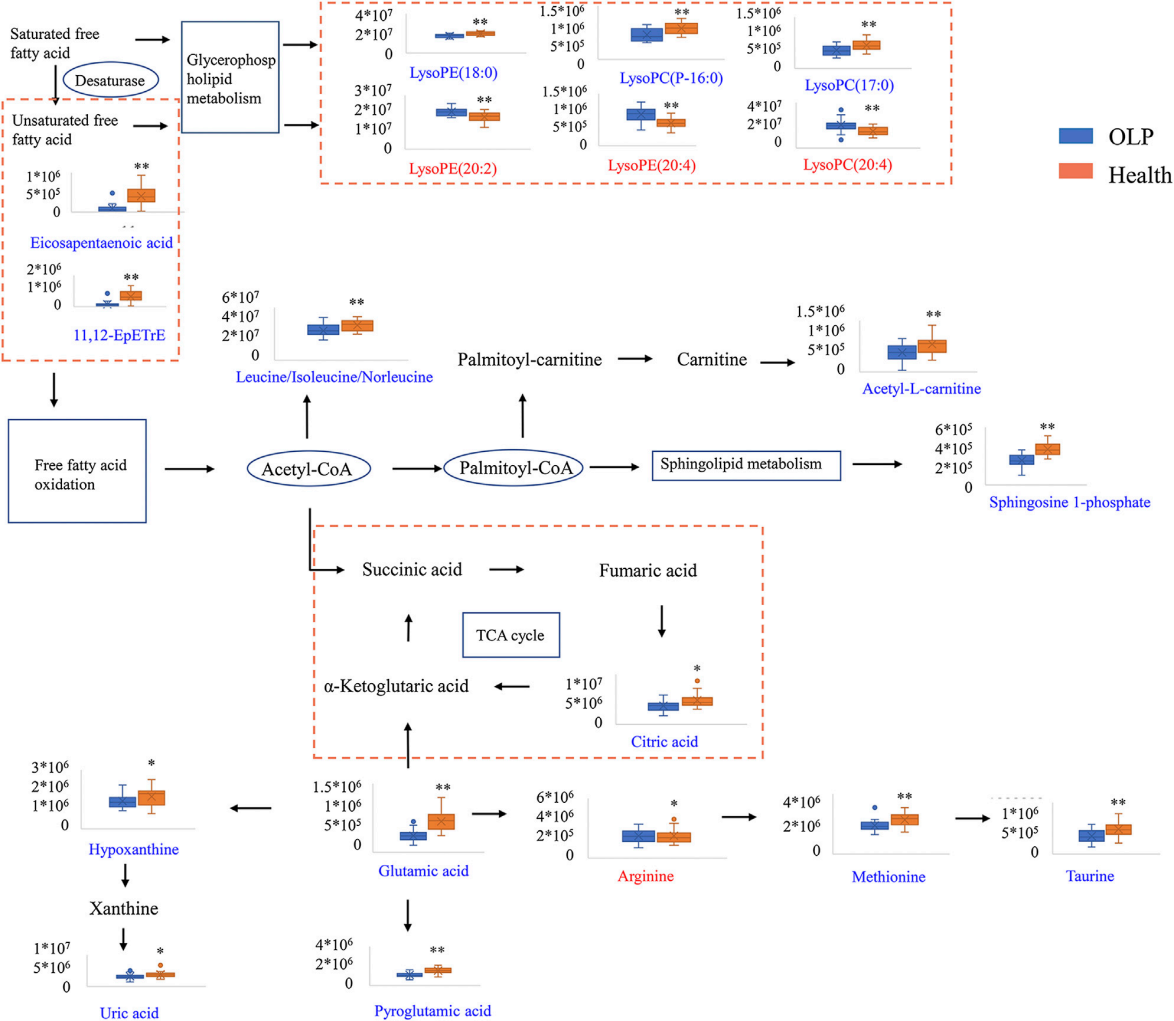
Metabolic pathway network based on the differential metabolites between OLP and healthy controls.

The perturbed pathways of metabolites occurred in the serum of patients with OLP were showed in Figure 5, including Taurine and hypotaurine metabolism, Alanine, aspartate and glutamate metabolism, Arginine and proline metabolism, Glutamine and glutamate metabolism and Aminoacyl-tRNA biosynthesis, etc. Based on the differential metabolites, the metabolic pathway network was mapped to display the metabolic disorders in patients with oral lichen planus (Figure 6). These results may provide an important basis for further studies of the pathogenic mechanism of OLP.

## Discussion

Currently, the identification of potential serum biomarkers for the detection of OLP remains challenged. Studies to date have been conducted on small population and small sample size.

In this study, we demonstrated an untargeted metabolomic evaluation for 115 OLP patients and 124 healthy controls based on UHPLC-Q-Orbitrap HRMS analysis. To our knowledge, this is the largest sample size of metabolomics research to date to investigate the potential biomarkers for early detection of OLP. Metabolic phenotypes revealed significant differences between OLP patients and healthy controls. We established a biomarkers panel for the detection of OLP, which consisted of Glutamic acid, LysoPE (18:0), and Taurine. This panel could be successfully used to reveal the change of metabolites and discriminate OLP from healthy individuals. The ability of this panel to diagnose OLP was excellent, with an accuracy of 87.1% in the test set.

OLP affects females more than males and commonly affects patients of middle age (Wagner et al., 2013). In our study, the mean age of OLP participants was 50.66, and the gender ratio between men and women is 48/67, that are basically consistent with the reported data. We collected the data of personal habits of each patients and healthy controls, and found that smoking was a strong risk factor for OLP. This result was supported by some previous studies (Villa and Gohel, 2014; Feng et al., 2015) indicating that subjects who has a habit of smoking were found to increase almost four-fold risk for oral malignant disorders. Another study showed that drinking alcohol on its own did not seem to increase the risk for oral malignant disorders, which is in consistent with our findings (Hassona et al., 2014). As for food eating habit, 44.3% OLP patients had a preference for very hot food before they got sick, while it was only 17.9% in healthy controls, which showed a significant difference between the two groups. But there was no significant difference for the preference for spicy food before they got sick. We also found that patients with OLP usually have less exercise and sleeping time than the healthy controls. Most patients claimed to have varying degrees of insomnia and we noticed that insomnia is more likely to lead to psychological problems, and people who are non-depressed subjects with insomnia have a two-fold risk to develop depression (Baglioni et al., 2011). Moreover, psychological factors are thought to play an important role in the pathogenesis of OLP (Soto Araya et al., 2004; Alrashdan et al., 2016). Extensive evidence has indicated that the depression is related to metabolic disturbance in glutamic metabolism (Moriguchi et al., 2019). We found decreased level in Glutamic acid concentration in the serum sample of patients.

Apoptotic cell death may be a contributory cause of basal cell destruction in oral lichen planus (Neppelberg et al., 2001). Phosphatidylcholine (PC) metabolism plays a significant role in the apoptotic program (Wright et al., 2004). In eukaryotic cellular membranes, the most abundant phospholipid moiety is phosphatidylcholine (Raetz, 1986) and apoptosis can be caused by inactivation of phosphatidylcholine biosynthesis (Cui et al., 1996). As a breakdown product of phosphatidylcholine (Zhao et al., 2019), we observed there were changes in four lysophosphatidylcholines including [LysoPC (17:0), LysoPC (P-16:0), LysoPC (16:1), and LysoPC (20:4)]. In addition, lipid-related molecules are often used as biomarkers for tumorigenesis (Defagó and Soria, 2010). Phosphatidylcholine might be the important contributory cause of apoptosis induction in oral precancerous lesions.

Taurine is widely distributed in human body, and it is closely associated with the development of immune system (Piao et al., 2019). It has been suggested that OLP patients might have some immune system dysfunction since the significantly decreased level of Taurine was observed on them. It might be a potential etiological factor of OLP.

Besides, patients with OLP have low defense to oxidative stress (Ergun et al., 2011). Low expression of Glutamic was found to be associated with oxidative stress (Wu et al., 2014). In this study, we found that the level of Glutamic was higher in healthy individuals, which indicated that oxidative stress may have potential biological effect in patients with OLP.

In summary, this study established a new method for the early detection of OLP, using UHPLC-Q-Orbitrap HRMS serum metabolomics analysis, which will help in understanding the pathological processes of OLP and identifying precancerous lesions in oral cavity.

## Data Availability Statement

The raw data supporting the conclusions of this article will be made available by the authors, without undue reservation.

## Author Contributions

X-SW and ZS contributed equally to this work. H-YZ and PX designed the research. X-SW, ZS, Z-SL, and Y-JY performed the experiments. X-SW, ZS, L-WL, and Q-ZD analyzed data. X-SW and ZS wrote the manuscript. L-WL and Q-ZD revised the manuscript. All authors read and approved the final manuscript.

## Funding

This work was supported by the National Natural Science Foundation of China (grant no. 81703666), and Kang Meng Medical Research Foundation (grant no. TB204022) by ZS in the author list.

## Conflict of Interest

The authors declare that the research was conducted in the absence of any commercial or financial relationships that could be construed as a potential conflict of interest.
