# Supplementary material for: Potential Metabolic Biomarkers for Early Detection of Oral Lichen Planus, a Precancerous Lesion
Source: Front Pharmacol. 2020 Nov 6;11:603899. doi: 10.3389/fphar.2020.603899 (PMC7677577; doi:10.3389/fphar.2020.603899)
Supplement: Supplementary file 1 [file DataSheet1_v1.PDF]

Supplementary Material

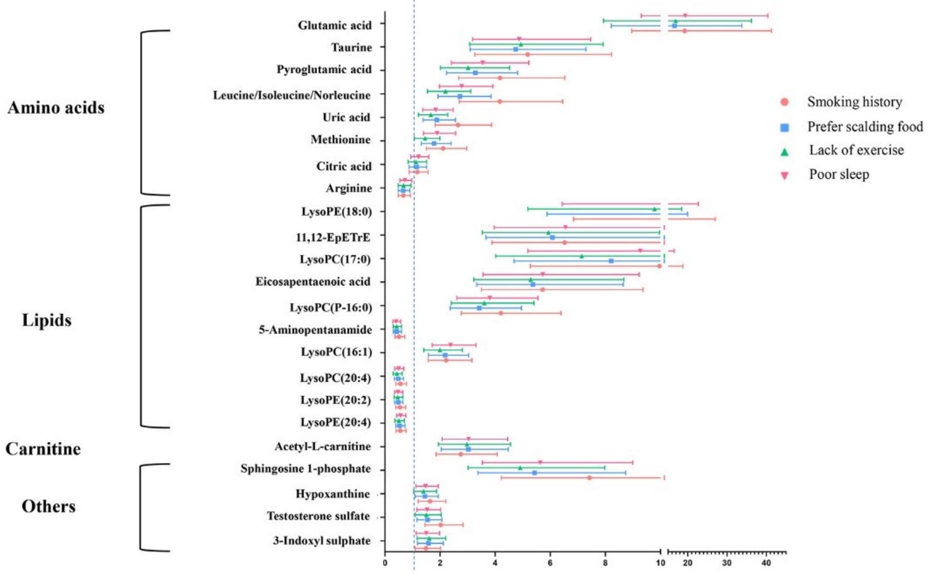

Supplementary Figure S1 ORs per SD increment (95% CI) of each metabolite based on results from multivariate-adjusted conditional logistic regression models.

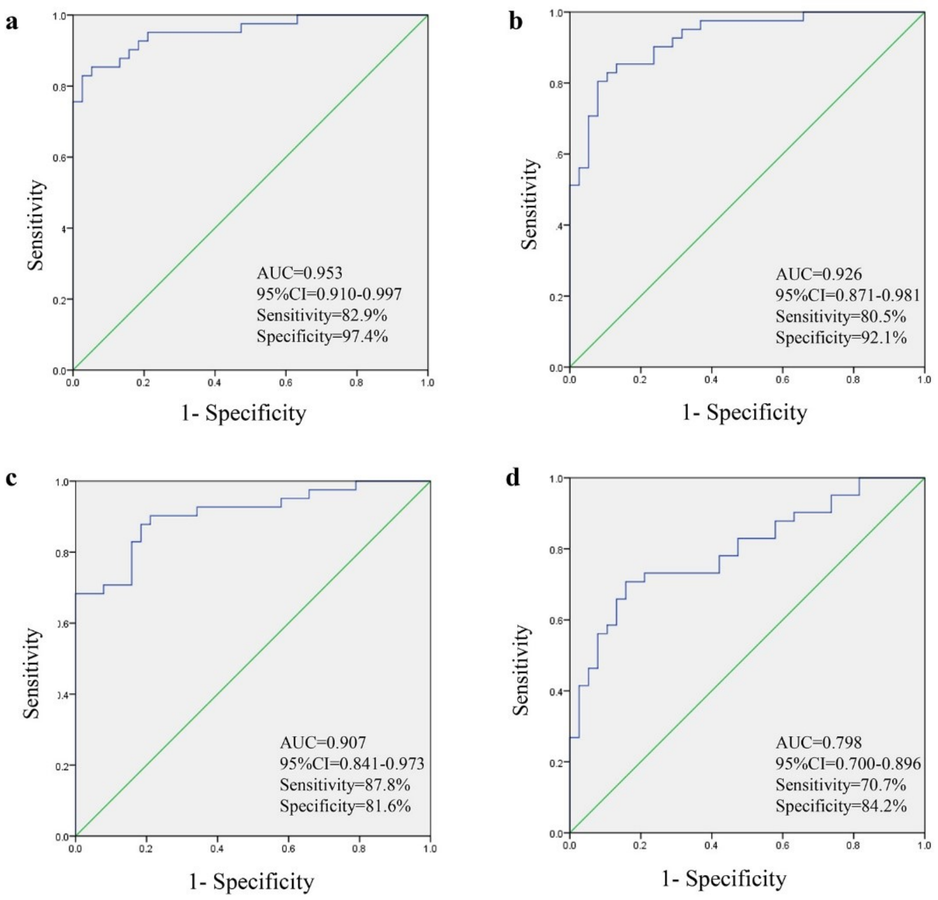

Supplementary Figure S2 The ROC curve with AUC, CI, sensitivity, specificity of (a) Diagnosis panel in training set, (b) Glutamic acid, (c) LysoPE(18:0) and (d) Taurine, respectively. AUC: Area under curve; CI: Confidence interval.

a

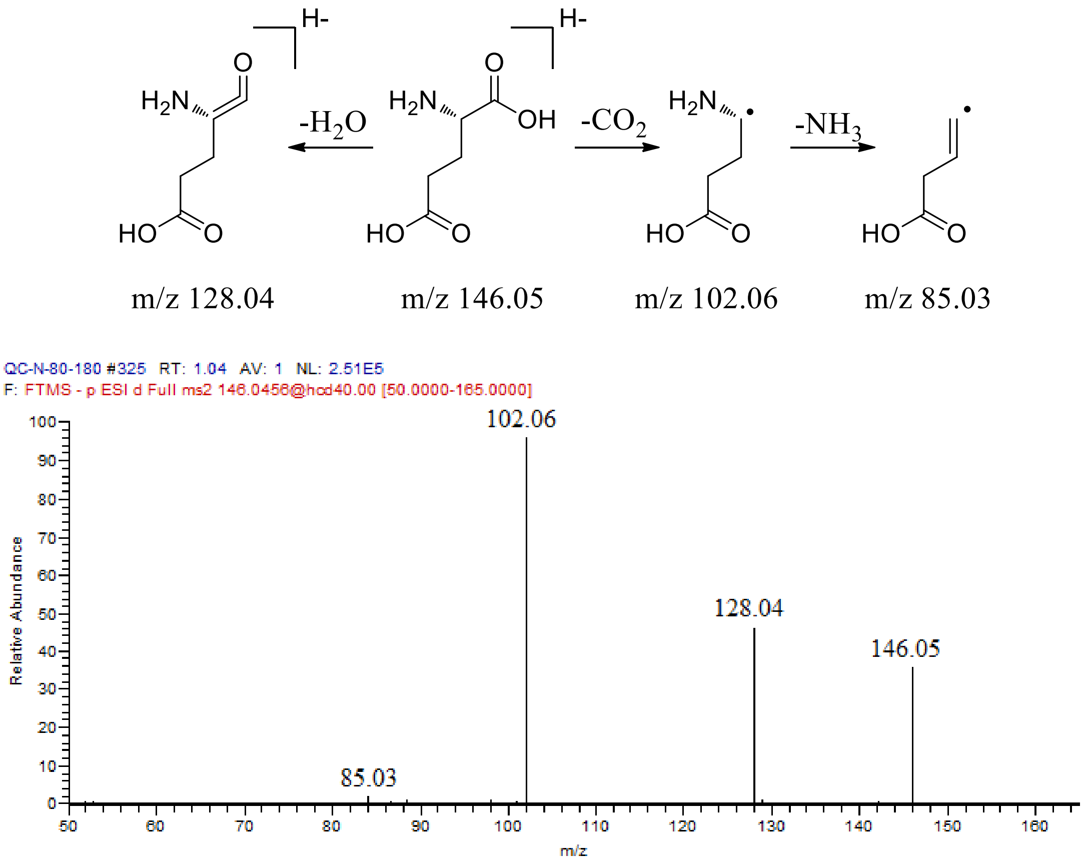

H-
-H_2O
-CO_2
-NH_3
m/z 128.04
m/z 146.05
m/z 102.06
m/z 85.03
QC-N-80-180 #325 RT: 1.04 AV: 1 NL: 2.51E5
F: FTMS - p ESI d Full ms2 146.0456@hcd40.00 [50.0000-165.0000]
102.06
128.04
146.05
85.03
Relative Abundance
m/z

b

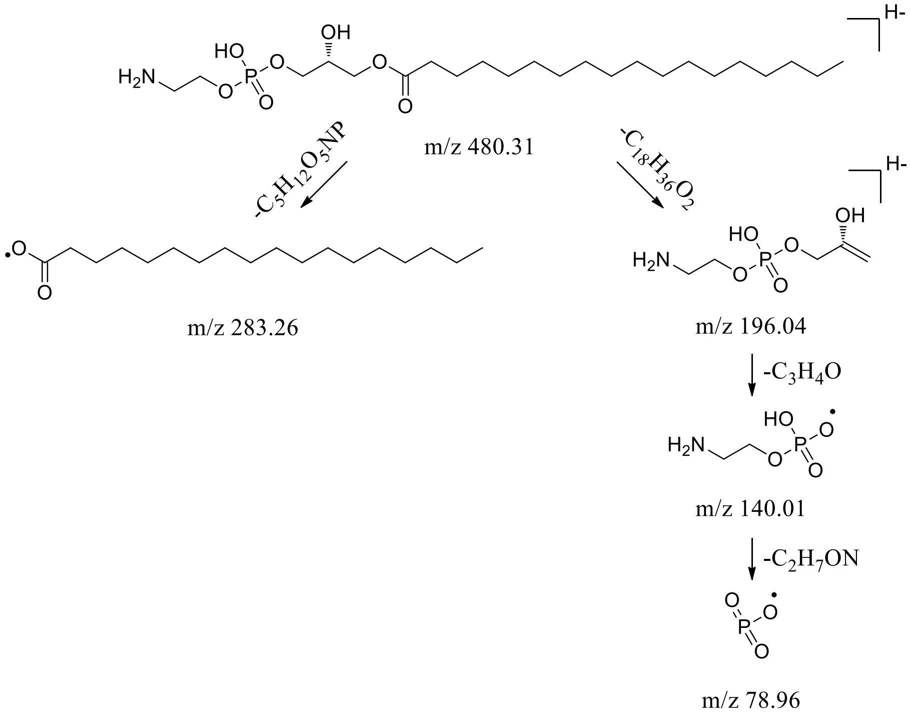

H-
m/z 480.31
-$C_5H_{12}O_5NP$
-$C_{18}H_{36}O_2$
m/z 283.26
m/z 196.04
-C_3H_4O
m/z 140.01
-C_2H_7ON
m/z 78.96

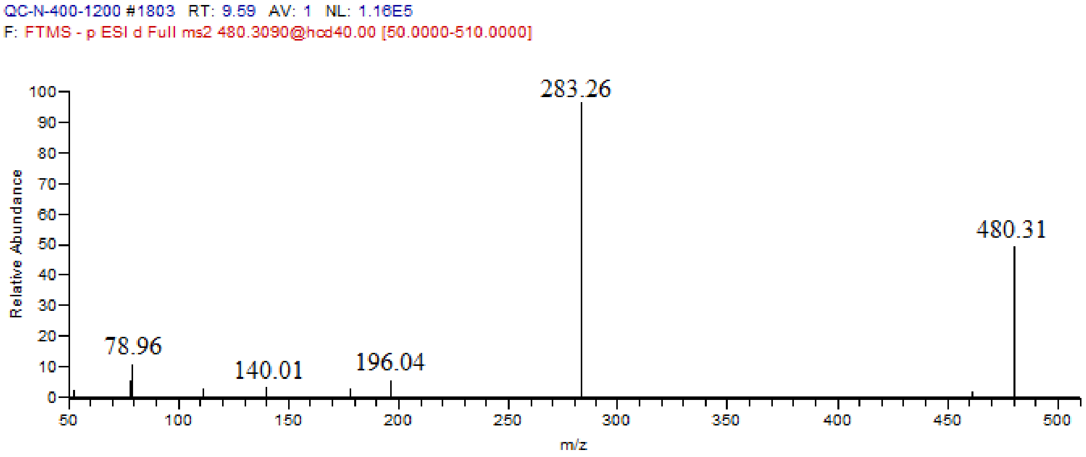

c

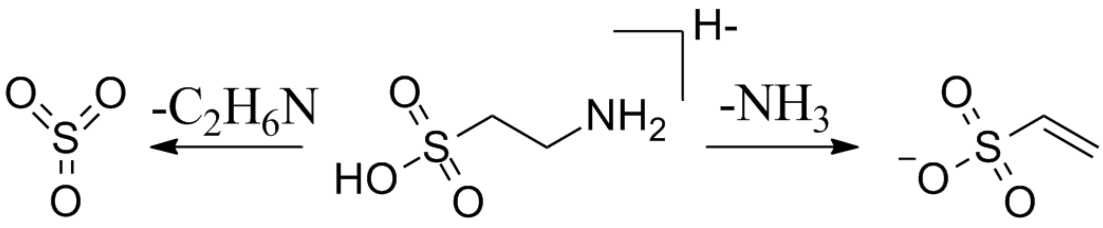

m/z 79.96 m/z 124.01 m/z 106.98

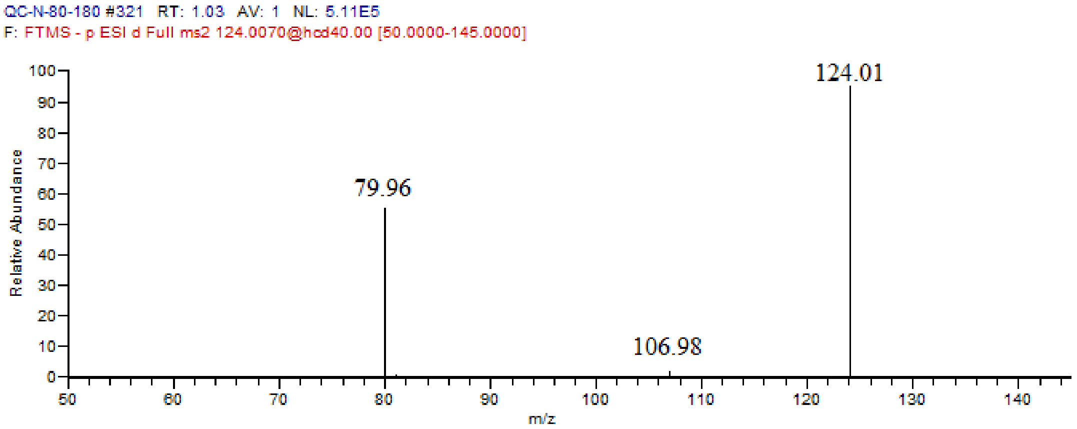

Supplementary Figure S3 The structures of three metabolic biomarkers and the mass fragmentation pathways. (a) Glutamic acid ($C_5H_9NO_4$) was observed under negative mode (m/z 146.05), and the fragment ions were 128.04 $[M\text{-}H\text{-}H_2O]^-$, 102.06 $[M\text{-}H\text{-}CO_2]^-$ and 85.03 $[M\text{-}H\text{-}CO_2\text{-}NH_3]^-$; (b) LysoPE(18:0) ($C_{23}H_{48}NO_7P$) was observed under negative mode (m/z 480.31), and the fragment ions were 283.26 $[M\text{-}H\text{-}C_5H_{12}O_5NP]^-$, 196.04 $[M\text{-}H\text{-}C_{18}H_{36}O_2]^-$, 140.01 $[M\text{-}H\text{-}C_{18}H_{36}O_2\text{-}C_3H_4O]^-$, 78.96 $[M\text{-}H\text{-}C_{18}H_{36}O_2\text{-}C_3H_4O\text{-}C_2H_7ON]^-$; (c) Taurine ($C_2H_7NO_3S$) was observed under negative mode (m/z 124.01), and the fragment ions were 106.98 $[M\text{-}H\text{-}NH_3]^-$, 79.96 $[M\text{-}H\text{-}C_2H_6N]^-$.
